# *Cdan1* Is Essential for Primitive Erythropoiesis

**DOI:** 10.3389/fphys.2021.685242

**Published:** 2021-06-21

**Authors:** Sharon Noy-Lotan, Orly Dgany, Nathaly Marcoux, Ayelet Atkins, Gary M. Kupfer, Linette Bosques, Christine Gottschalk, Orna Steinberg-Shemer, Benny Motro, Hannah Tamary

**Affiliations:** ^1^Molecular Pediatric Hematology Laboratory, Schneider Children’s Medical Center of Israel, Petach Tikva, Israel; ^2^Felsenstein Medical Research Center, Tel Aviv University, Tel Aviv, Israel; ^3^The Institute for Nanotechnology and Advanced Materials, Bar-Ilan University, Ramt Gan, Israel; ^4^Department of Oncology and Pediatrics, Lombardi Comprehensive Cancer Center, Georgetown University, Washington, DC, United States; ^5^Department of Cell Biology, Yale School of Management, Yale University, New Haven, CT, United States; ^6^Department of Hematology, Oncology, Immunology, and Rheumatology, University Hospital Tübingen, Tübingen, Germany; ^7^The Rina Zaizov Hematology-Oncology Division, Schneider Children’s Medical Center of Israel, Petach Tikva, Israel; ^8^Sackler Faculty of Medicine, Tel Aviv University, Tel Aviv, Israel; ^9^The Mina and Everard Goodman Faculty of Life Sciences, Bar-Ilan University, Ramat Gan, Israel

**Keywords:** primitive erythropoiesis, Codanin-1, mice, zebrafish, Cdan1

## Abstract

Congenital dyserythropoietic anemia type I (CDA I) is an autosomal recessive disease characterized by moderate to severe macrocytic anemia and pathognomonic morphologic abnormalities of the erythroid precursors, including spongy heterochromatin. The disease is mainly caused by mutations in *CDAN1* (encoding for Codanin-1). No patients with homozygous null type mutations have been described, and mouse null mutants die during early embryogenesis prior to the initiation of erythropoiesis. The cellular functions of Codanin-1 and the erythroid specificity of the phenotype remain elusive. To investigate the role of Codanin-1 in erythropoiesis, we crossed mice carrying the *Cdan1* floxed allele (Cdan^*fl/fl*^) with mice expressing Cre-recombinase under regulation of the erythropoietin receptor promoter (ErGFPcre). The resulting Cdan^Δ*Ery*^ transgenic embryos died at mid-gestation (E12.5–E13.5) from severe anemia, with very low numbers of circulating erythroblast. Transmission electron microscopy studies of primitive erythroblasts (E9.5) revealed the pathognomonic spongy heterochromatin. The morphology of Cdan^Δ*Ery*^ primitive erythroblasts demonstrated progressive development of dyserythropoiesis. Annexin V staining showed increases in both early and late-apoptotic erythroblasts compared to controls. Flow cytometry studies using the erythroid-specific cell-surface markers CD71 and Ter119 demonstrated that Cdan^Δ*Ery*^ erythroid progenitors do not undergo the semi-synchronous maturation characteristic of primitive erythroblasts. Gene expression studies aimed to evaluate the effect of *Cdan1* depletion on erythropoiesis revealed a delay of ζ to α globin switch compared to controls. We also found increased expression of *Gata2*, *Pu.1*, and *Runx1*, which are known to inhibit terminal erythroid differentiation. Consistent with this data, our zebrafish model showed increased *gata2* expression upon *cdan1* knockdown. In summary, we demonstrated for the first time that *Cdan1* is required for primitive erythropoiesis, while providing two experimental models for studying the role of Codanin-1 in erythropoiesis and in the pathogenesis of CDA type I.

## Introduction

The congenital dyserythropoietic anemias (CDAs) are a group of rare inherited disorders that are characterized by impaired erythropoiesis and distinguishing cytopathology of the erythroid cells. CDAs have been classified into four types (I-IV) (renella Delaunay and Iolascon, 1999; Heimpel, 2004; Arnaud et al., 2010; Iolascon et al., 2013). CDA-I is an autosomal recessive disease associated with moderate-to-severe macrocytic anemia and occasional bone abnormalities, particularly syndactyly of fingers and toes, and the absence of nails (Tamary et al., 1996, 2005; Delaunay and Iolascon, 1999; Heimpel et al., 2006; Niss et al., 2021). Bone marrow aspirates reveal binuclear intermediate and late erythroid precursors, and internuclear chromatin bridges. Ultra-structural erythroid features include “spongy” heterochromatin (“Swiss cheese type”), enlargement of the nuclear pores and invagination of the nuclear membrane. For some patients, administration of interferon-α (INF-α) improves anemia and normalizes erythroid morphology (Lavabre-Bertrand et al., 2004; Abu-Quider et al., 2020), although this treatment has significant toxicities.

In 2002, we identified *CDAN1* as the causative gene for CDA-I (Dgany et al., 2002). Bi−allelic mutations in *CDAN1* occur in ∼80% of individuals with CDA-I. This ubiquitously expressed gene spans 28 exons and encodes the protein Codanin−1, a ∼134 kD, highly evolutionarily conserved protein (Dgany et al., 2002). The *Drosophila* homolog, discs lost (Dlt), is required for cell survival and cell−cycle progression (Pielage et al., 2003). The expression patterns and function of zebrafish *cdan1* ortholog remain unknown.

Codanin-1 is a direct transcriptional target of the E2F1 transcription factor; its expression is cell-cycle regulated (Noy-Lotan et al., 2009). The Phyre2 alignment program (Kelley et al., 2015), which identifies 3D structurally homologous proteins, revealed high similarity of Codanin-1 to CNOT1. The latter is a conserved protein that serves as a scaffold for proteins involved in mRNA stability and transcriptional control (Swickley et al., 2020).

*CDIN1* (previously designated C15orf41), a second causative gene for CDAI, was identified in 2013 (Babbs et al., 2013). Bi−allelic mutations in *CDIN1* are found in ∼10% of individuals with CDA-I (Babbs et al., 2013). The function of the protein is not known, but its sequence suggests a role as an endonuclease, presumably related to Holliday junction resolvases (Babbs et al., 2013). Codanin-1 was recently shown to interact directly with CDIN1. When overexpressed, Codanin-1 holds and localizes CDIN1 within the cytosol, possibly serving as a scaffold protein (Shroff et al., 2020; Swickley et al., 2020). Whether this phenomenon has a functional relevance in some physiological circumstances is not yet clear. Both proteins were found to be enriched in the nucleolus (Scott et al., 2020; Olijnik et al., 2021).

Both CDIN1 and Codanin−1 interact with the histone chaperone Asf1b (anti silencing factor 1b) (Ewing et al., 2007; Ask et al., 2012). Asf1 is a conserved histone H3–H4 chaperone that has been implicated in a variety of aspects of nucleosome-assembly, eviction and exchange. Codanin−1 sequesters Asf1 in the cytoplasm, thereby negatively regulating the supply of histone-bound Asf1 to the nucleus. This suggests that Codanin-1 guards a limiting step in chromatin replication (Ask et al., 2012).

To date, no homozygotes for a null type *CDAN1* mutation have been identified in humans. This suggests that a complete lack of Codanin-1 protein is lethal (Roy and Babbs, 2019). In agreement, mice homozygous for null *Cdan1* allele die at an early stage of embryonic development (i.e., ∼6.5 dpc), even prior to the onset of erythropoiesis (Renella et al., 2011). This suggests a developmental role for Codanin-1. However, the basis for the high sensitivity of the erythroid lineage to lower activity of Codanin-1 remains to be elucidated.

During erythropoiesis, hematopoietic stem cells are committed to erythroid progenitors, which subsequently undergo terminal erythroid differentiation to produce mature red blood cells. Erythroid maturation is tightly linked with cell-cycle progression: proerythroblasts undergo 4–5 cell divisions in which they decrease in cell size and undergo chromatin condensation and hemoglobinization. This leads to their enucleation and the expulsion of other organelles. The process is regulated at a number of levels by multiple proteins and miRNAs (Hattangadi et al., 2011; Hu et al., 2013).

Erythropoiesis entails two developmental phases: primitive (embryonic) and definitive (fetal and adult). In mice, primitive erythropoiesis initiates in the yolk sac at embryonic day 7.5 (E7.5), and definitive erythropoiesis begins in the fetal liver by E11.5. The primitive erythroid lineage in mammalian embryos is characterized by a transient wave of lineage-committed progenitors that emerge from the yolk sac to generate precursors. These cells synchronously mature in the bloodstream, progressively lose proliferative capacity, decrease in cell size, accumulate hemoglobin, and undergo nuclear condensation and enucleation (Kingsley et al., 2006; Palis, 2014). A second wave of embryonal hematopoiesis known as erythroid-myeloid progenitors (EMP) originates in the yolk sac at day E8.25, moves to the blood stream and then to the fetal liver where it generates the first circulating definitive hematopoiesis (Palis et al., 1999, 2001). Definitive permanent erythropoiesis occurs in the fetal liver and subsequently in the bone marrow. Both primitive and definitive erythropoiesis, are characterized by the progressive differentiation of cells from progenitors to erythroblast precursors, and to red blood cells.

Investigation of the pathogenesis of CDA-I has been hampered by the lack of an appropriate *in vivo* model that recapitulates key features of the disease. *Cdan1*^Δ/Δ^ knockout mice die before the onset of erythropoiesis (Renella et al., 2011); while c57bl/6 Cdan1 (R1054W) knock-in mice harboring the most common Bedouin founder mutation (*Cdan1*^*R*1054W/R1054W^), are viable and fertile, and do not show any erythroid defects (GMK, unpublished data).

In this work, we assessed the role of *Cdan1* in primitive erythropoiesis, by studying a *Cdan1* erythroid-conditional knockout mouse and zebrafish models. We conclude that *Cdan1* is necessary for primitive erythropoiesis and that these models can be further used to study the role of Codanin-1 in erythropoiesis and in the pathogenesis of CDA- I.

## Materials and Methods

### Animals

To create a conditional targeted allele, we used a *Cdan1* knockout-first ES cell line (generated by KOMP- the Knock-Out Mouse Project, designated Cdan1^*tm*1a(KOMP)Wtsi^ (EUCOMM). This allele was transformed to a *CDAN1*-floxed allele by activation of Flippase. This generated *Cdan1*^*f*/f^ mice with LoxP-sites flanking exons 7–10 of *Cdan1* (Supplementary Figure 1). These were crossed with erythropoietin receptor Cre recombinase transgenic mice (Heinrich et al., 2004), to generate *Cdan*^Δ*Ery*^ mice with erythroid-specific deletion of *Cdan1.* Excision of exon 7–10 by Cre should result in a truncated protein that misses the conserved central region and thus presumably creates a null allele. In addition, the deletion of exon 7–10 should yield premature protein termination in exon 11. The following primers were used to genotype mice: for Cdan1^*flox*^: 5′-GTTTCGTAGCATCCATGATTGC-3′ and 5′-GCACAGCAGCCGCTCATTCTCAG-3′; for iCre: 5′-AGATGCCAGGACATCAGGAACCTG-3′ and 5′-ATCAGCC ACACCAGACACAGAGATC-3′; for *Cdan*^Δ^: 5′-GTTTCGTA GCATCCATGATTGC-3′ and 5′-TCTGAGATGTCCGAAGGG AG-3′. All animal experiments were approved by the Animal Ethics Committee of the Faculty of Medicine in Tel Aviv University (Protocol Number 01-16-019).

All zebrafish experiments described in the present study were conducted on embryos younger than 3 days post-fertilization (dpf). Zebrafish were maintained according to standard protocols and handled in accordance with European Union animal protection Directive 2010/63/EU and approved by the local government (husbandry permit 35/9185.46/Uni TÜ).

### Analysis of *Cdan*^Δ*Ery*^ Embryos and Peripheral Blood

Timed pregnant females were euthanized by asphyxiation with CO_2_ according to institutional guidelines. Uteri were removed and washed with PBS, and embryos were dissected free of decidual tissue in wash buffer (PBS+5% FBS). Individual embryos were transferred to clean dishes containing heparinized wash buffer (PBS + 5%FBS + 0.2 U/ml heparin) and exsanguinated by decapitating and by severing the umbilical vessels. We then allowed the blood to drain into a well of 12-well plate containing 0.5 ml of heparinized wash buffer. For red cell counts, the collected cells were stained with trypan blue for dead cell exclusion and scored using a hemocytometer.

Embryos were dissected under a Nikon SMZ745T dissecting microscope (Nikon, Tokyo, Japan). Photographs were taken with a DeltaPix Camera (Invenio 5SCIII) and acquired using DeltaPix InSight software (DeltaPix, Smoerum, Denmark).

For whole embryo benzidine staining, embryos were incubated for 15 min in 12% glacial acetic acid containing 0.4% benzidine dihydrochloride (Sigma B3383) supplemented with 0.3% hydrogen peroxide (H_2_O_2_) at room temperature.

### Cytospin Analyses

Peripheral blood cells from E9.5 to E12.5 embryos were collected as detailed above and cytospun onto glass slides at 400 g for 10 min (Cellspin^®^ III, Tharmac). Slides were stained with Modified Wright’s Stain on a Siemens hematek 3000 system. The slides were viewed using an Olympus BX52 microscope (Olympus, Tokyo, Japan) with an Olympus UPlanApo 100x/1.35 NA Oil Iris objective (1,000 × total magnification). Micrographs were taken with an Olympus DP72 digital camera and with CellSens image acquisition software (Olympus, Tokyo, Japan).

### Flow Cytometry

To monitor erythroid differentiation status, circulating E9.5–E11.5 erythroblasts were stained with PE-conjugated anti-Ter119 and APC-conjugated anti-CD71 (Biolegend). Antibodies were used at 1:200. Dead cells were excluded with 7-AAD staining. For analyses of apoptosis, erythroblasts were co-stained with Ter119, CD71, 7AAD and Annexin V (Biolegend). All the experiments were performed on a FACSGallius flow cytometer (Beckman Coulter). Data were acquired and analyzed using Kaluza^®^ for Gallios Acquisition Software (Beckman Coulter). Values represent averages across biological replicates ± SEM. The student’s paired *t*-test was used to determine significance (*p* < 0.05).

### Cell Cycle Analysis

For cell cycle analyses, BrdU incorporation was done *ex vivo* as previously described (Malik et al., 2013). Briefly, E9.5 circulating erythroblasts were cultured for 90 min in erythroid differentiation media (IMDM, 20% FBS (Biological Industries Inc., Israel), 10% PFHM-II (Invitrogen), 2 mM L- glutamine, 150 μM MTG (Sigma), and 1 U/mL recombinant human EPO (PeproTech Asia), supplemented with 10 μM bromodeoxyuridine (BrdU). BrdU incorporation was detected using a FITC BrdU flow kit (BD Biosciences), according to the manufacturer’s instructions, on a FACSGallius flow cytometer (Beckman Coulter). Data were acquired and analyzed using Kaluza^®^ for Gallios Acquisition Software (Beckman Coulter).

### RNA Extraction and qPCR

E9.5–E11.5 circulating erythroblasts were collected as detailed above and RNA was extracted using Qiagen RNAeasy kit (Qiagen, United States). cDNA was synthesized with the Maxima cDNA synthesis kit (Thermo Fisher Scientific, United States). qPCR was performed on an Illumina ECO Real-Time PCR system (Illumina, United States). The primers used are detailed in Supplementary Table 1. The results were normalized to expression levels of the mouse *Rplp0* and zebrafish *bactin* house-keeping genes, respectively.

### Transmission Electron Microscopy

For preparation of cellular transmission electron microscopy (TEM) samples, peripheral blood E9.5–E11.5 cells were harvested as described above. The cells were fixed for 2 h in Karnovsky-fixative [2.5% glutaraldehyde with 2.5% paraformaldehyde in a 0.1M sodium cacodylate buffer (pH 7.4)] and washed with 0.1 M sodium cacodylate buffer. The cells were post-fixed in 1% OsO_4_, 0.5% K_2_Cr_2_O_7_, 0.5% K_4_[Fe(CN)_6_] in 0.1M cacodylate-buffer (pH 7.4) for 1 h at room temperature, then washed twice with 0.1M cacodylate-buffer, and subsequently rinsed with DDW three times. Cells were then stained with 2% uranyl-acetate for 1 h, washed with DDW, and dehydrated in ethanol and embedded in EMbed 812 (EMS, United States). The resin was polymerized at 60°C for 24 h. Ultrathin sections (70–90 nm) were obtained with a Leica EM UC7 Ultramicrotome, then analyzed in a Tecnai G-12 Spirit electron microscope (FEI, Japan).

### Morpholino Injection

Two morpholinos (MO) (GeneTools) targeting the zebrafish *cdan1* gene were used in this study (MO1: CAGGTGCTGTAT TAACTCTTACATG and MO2: ATCACCTACTCCTCACTTAC ATTGG). The efficiency of MOs was assessed by qPCR. A scrambled MO (CCTCTTACCTCAGTTACAATTTATA) was used as a negative control. Each MO was injected at 400–600 μM into one-cell stage zebrafish embryos.

### Whole Mount *in situ* Hybridization (WISH)

Zebrafish embryos were fixed in 4% PFA in 2xPBS, 0.1% Tween-20 for 24 h at 4°C. WISH was performed as described previously (Kuri et al., 2017) using digoxigenin-labeled RNA antisense probes, which are listed in Supplementary Table 2.

### *o*-Dianisidine Staining

Five hundred microliter of *o*-dianisidine solution (0.6 mg/ml *o*-dianisidine, 0.01 M sodium acetate (pH = 4.5), 0.65% H_2_O_2_, and 40% ethanol) was added to live dechorionated embryos. After 15–45 min of incubation in the dark, the samples were 3x washed with dH_2_O and then fixed in 4% PFA for 1 h at room temperature. Images were taken after several washings with PBS and 0.1% Tween-20.

## Results

### Erythroid-Specific Ablation of *Cdan1* Recapitulates CDA Type I Phenotype

To investigate the specific role of Codanin-1 in erythropoiesis, we crossed *Cdan1*^*flox/flox*^ mice to ErGFPcre mice (Heinrich et al., 2004). In ErGFPcre mice, Cre mediated recombination of the floxed alleles is regulated by the endogenous erythropoietin receptor (Supplementary Figure 1). To validate Cre recombination, we genotyped erythroid cells from heterozygous *Cdan1*^*flox/*+^;ErGFPcre mice, and found positive erythroid-specific Cre excision of exons 7–10 of *Cdan1* (Supplementary Figure 2A). *EpoR*-dependent Cre expression presumably begins at E7.5 and increases at E8.5 (McGann et al., 1997). Accordingly, we found that E10.5 erythroblasts from Cdan^*flox/flox*^; ErGFPcre (Cdan^Δ*Ery*^) embryos expressed the GFP-Cre fusion protein (Supplementary Figure 2B). Analysis of peripheral blood from E9.5 Cdan^Δ*Ery*^ embryos demonstrated a markedly reduced (to ∼20%) level of *Cdan1* expression (Supplementary Figure 2C).

**FIGURE 1:**
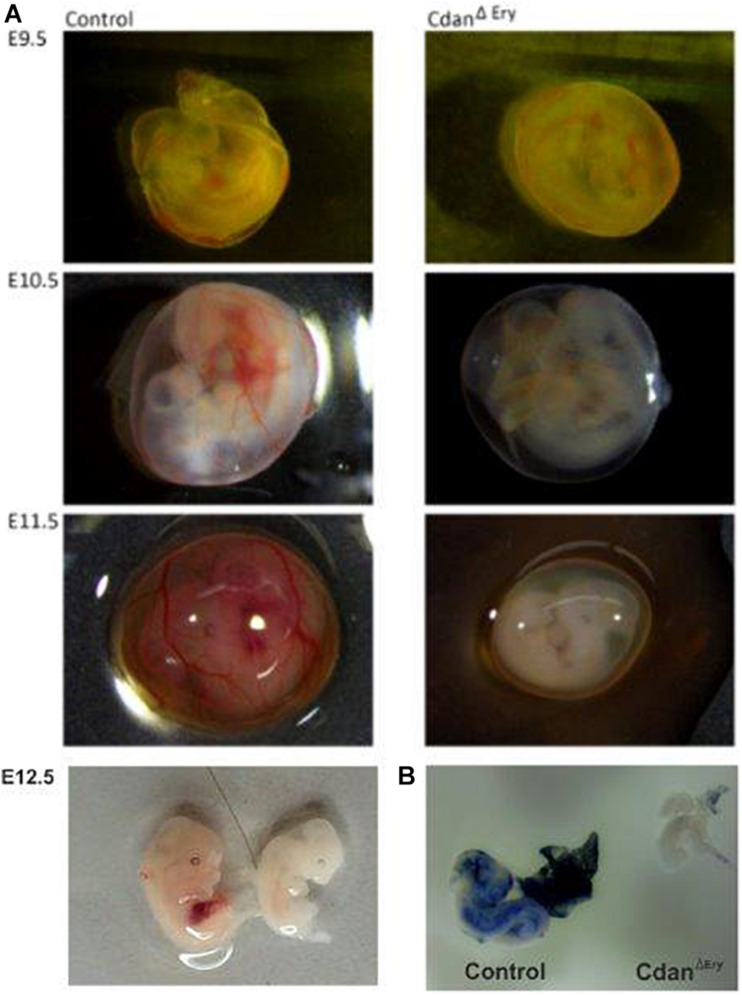
Cdan^Δ*Ery*^ embryos die in-utero between E12.5 and E13.5 from severe anemia. **(A)** Cdan^Δ*Ery*^ embryos were extracted and photographed at different dpc. From E10.5 and on, Cdan^Δ*Ery*^ embryos and Yolk Sacs (right column) are very pale with no visible vasculature or Fetal Liver. **(B)** Benzidine staining of E10.5 Cdan^Δ*Ery*^ embryo (right) Vs. littermate control (left). Mutant embryo and Yolk sac stained very lightly and are notably smaller.

Crossing Cdan^*flox/flox*^ with Cdan^*flox/*+^;ErGFPcre mice did not yield any live offspring with a Cdan^Δ*Ery*^ genotype (Table 1). Careful examination of timed embryos revealed that Cdan^Δ*Ery*^ transgenics appeared grossly normal at E9.5, but pale and apparently anemic from day E10.5 and onwards (Figure 1A). No mutant embryos with a visible heartbeat were observed at E13.5. This suggests mutants die at mid-gestation (E12.5–E13.5) probably due to severe anemia, as evident by complete lack of benzidine staining of mutant whole embryos (Figure 1B).

**TABLE 1:** The number of Cdan^Δ*Ery*^ embryos generated from Cdan^*f/f*^ X Cdan^*f/*+;ErGFPcre^ crossing.

DNA source	Total embryos	Expected live Cdan^Δ*Ery*^	Observed live Cdan^Δ*Ery*^	Observed dead Cdan^Δ*Ery*^
E8.5	23	6	6	0
E9.5	86	22	22	0
E10.5	71	18	16	0
E11.5	59	15	17	0
E12.5	17	4	4	1
E13.5	8	2	0	3
3 weeks tail	213	53	0	–

**FIGURE 2:**
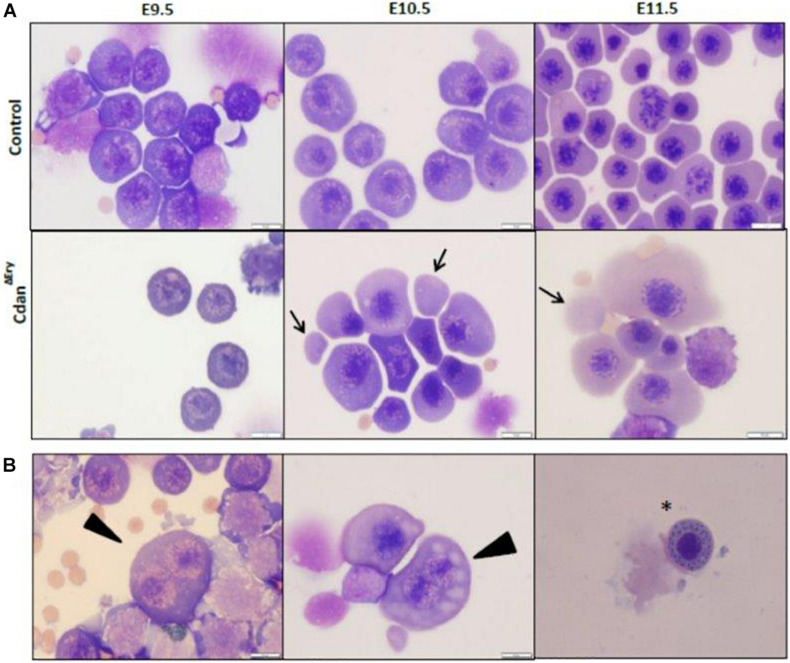
Erythroid-specific knockout of *Cdan1* recapitulates the CDA-I phenotype. **(A)** Morphology of Cdan^Δ*Ery*^ circulating erythroblasts, compared to littermate controls. Cytospins were prepared from blood samples collected from E9.5 to E11.5 yolk sacs and embryos. The arrows mark primitive enucleated cells. Scale bar = 10 μm. **(B)** Cdan^Δ*Ery*^ erythroblasts show aberrant morphology including binuclear cells (arrowhead) and basophilic stippling (asterisk). Scale bar = 10 μm.

**FIGURE 3:**
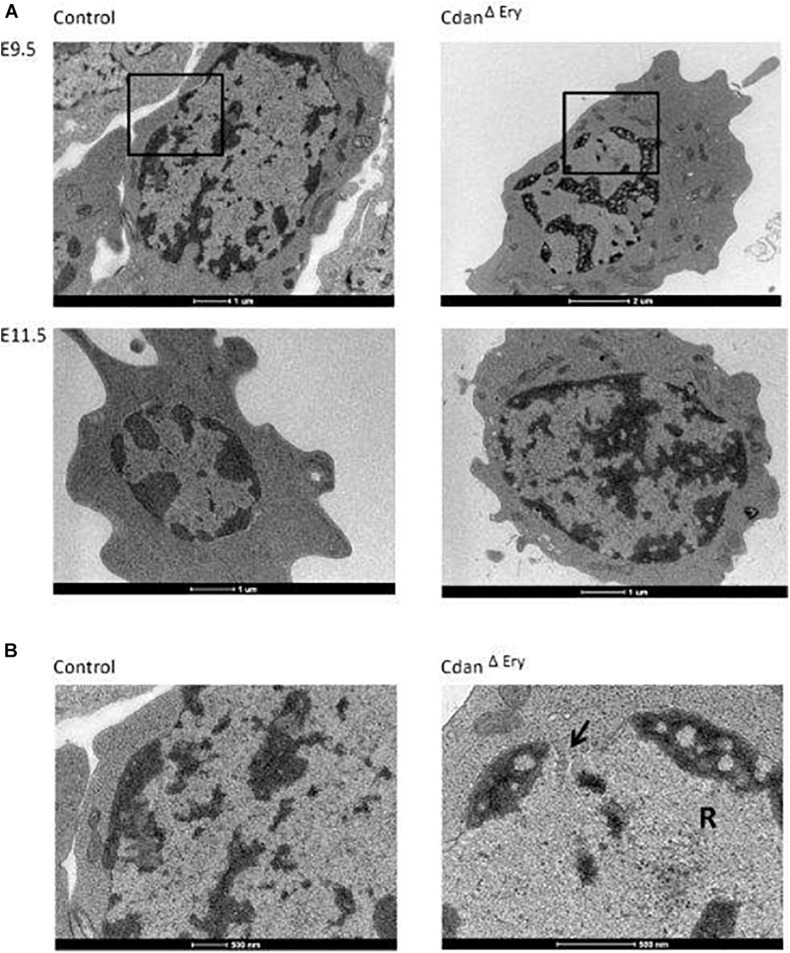
Cdan^Δ*Ery*^ erythroblasts display the pathognomonic spongy heterochromatin of CDA type I. **(A)** TEM of E9.5 (upper panel) and E11.5 (lower panel) Cdan^Δ*Ery*^ erythroblasts compared to littermate controls. Magnification: X6000-9900. Scale bar: 1–2 μm. **(B)** High power magnification (X20,000-43,000) of E9.5 erythroblasts in A, showing the normal appearance of heterochromatin and the intact nuclear membrane in control erythroblast (left), compared to Cdan^Δ*Ery*^ erythroblast with spongy heterochromatin, dilated membrane pore (arrow) and ribosomes (R) inside the nucleus. Scale bar: 500 nm.

Bone marrow aspirates of CDA-I patients show a range of abnormalities, including bi-nuclear cells, chromatin bridges, megaloblastic cells, karyorrhexis and basophilic stippling (Heimpel et al., 2010). To examine the morphology of Cdan^Δ*Ery*^ erythroblasts, embryonic peripheral blood collected at E9.5–E12.5 was cytospun onto slides and stained with Modified Wright’s stain (Figure 2A). Careful examination demonstrated aberrant morphological features, similar to those observed in individuals with CDA-I (Figure 2B). Spongy or “Swiss-cheese” like heterochromatin is considered the pathognomonic feature of CDA-I. TEM analysis revealed that Cdan^Δ*Ery*^ erythroblasts exhibit this heterochromatin abnormality, which is characteristic of bone marrow erythroid progenitors of individuals with CDAI (Figure 3A). In addition to the spongy heterochromatin, Cdan^Δ*Ery*^ erythroblasts exhibited disruption of the nuclear membrane with cytoplasmic invagination (Figure 3B). These results demonstrate that erythroid-specific deletion of *Cdan1* recapitulates the CDA-I phenotype.

### Loss of *Cdan1* Impairs Proliferation and Survival of Primitive Erythroblasts

**FIGURE 4:**
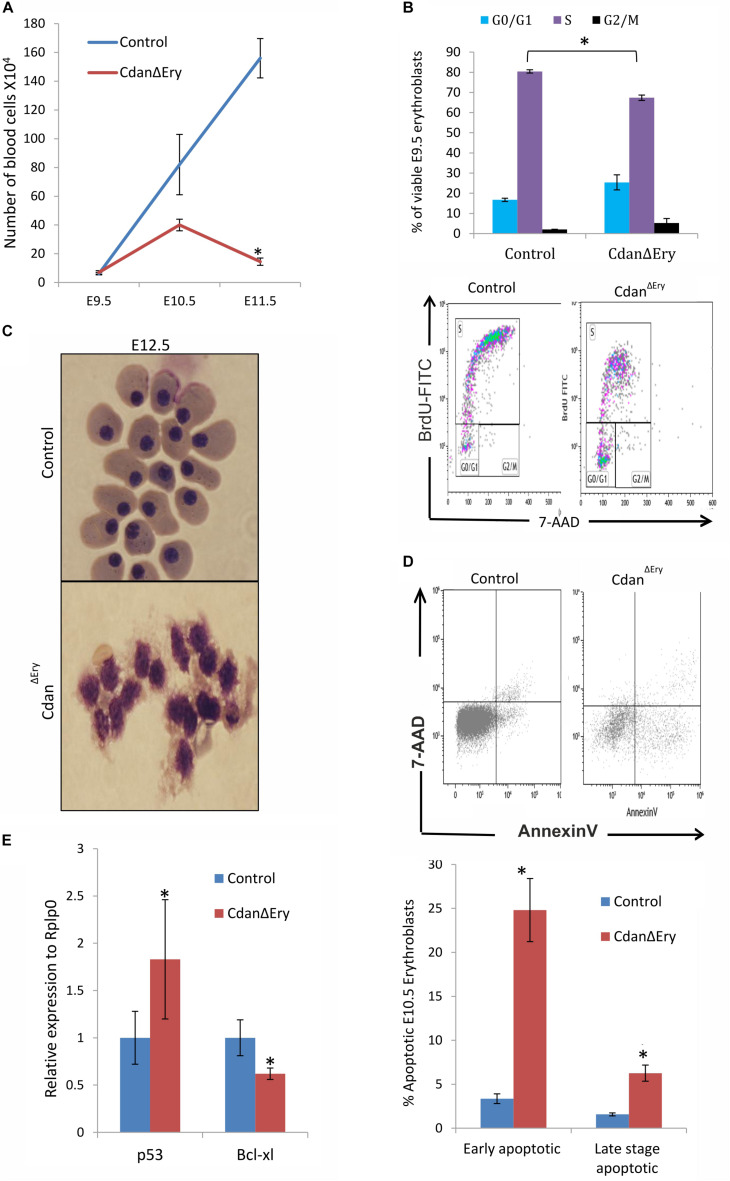
*Cdan1* depletion impairs cell cycle progression and increases cell death. **(A)** Number of circulating primitive erythroid cells collected from E9.5 to E11.5 control and Cdan^Δ*Ery*^ embryos. Values represent mean ± Standard error. Cdan^Δ*Ery*^: *n* = 3; Control: *n* = 9. *p < 0.002 compared to controls. **(B)** Cdan^Δ*Ery*^ E9.5 erythroblasts show aberrant G1-to-S progression. The percentage of cells in G0/G1, S and G2/M phases of the cell cycle in wild-type (control) and Cdan^Δ*Ery*^ E9.5 erythroblasts gated for the CD71^+^/Ter119^+^ population. Cdan^Δ*Ery*^ *n* = 3; control *n* = 12. Error bars indicate standard error. *p < 0.02 compared to wild-type. **(C)** Morphology of E12.5 Cdan^Δ*Ery*^ circulating erythroblasts, compared to littermate controls. **(D)** E10.5 peripheral blood cells were gated for CD71+/Ter119+ erythroblasts, and analyzed for AnnexinV/7AAD staining to assess for apoptosis (Representative flow plots are shown in upper panel). Early apoptotic cells are AnnexinV-positive/7-AAD-negative; Late apoptotic cells are double positive. Data presented as mean ± SEM (n = 3-4 for each genotype). *p < 0.005. **(E)** *Cdan1* ablation decreases Bcl-xl expression and increases p53 levels. qRT-PCR was performed with RNA extracted from peripheral blood cells at E11.5 and normalized to Rplp0. Relative expression data are presented as mean ± SEM (*n* = 4–6 for each genotype). Student’s *t*-test was applied for statistical analysis. Asterisks indicate significant difference from the Control. *p < 0.05.

Maturation of primitive erythroid precursors is associated with expansion of erythroblast numbers through a limited set of symmetric cell divisions. Erythroblast numbers were shown to increase dramatically (∼100-fold) between E8.5 and E10.5 of mouse gestation (Fraser et al., 2007; Palis, 2014). This supports the growing oxygen demand of the developing embryo. To examine whether impaired proliferation contributes to the visible anemia of Cdan^Δ*Ery*^ embryos, we quantified circulating primitive erythroblasts from E9.5 to E11.5 Cdan^Δ*Ery*^ embryos and control littermates (Figure 4A). The number of cells in the controls increased by ∼10-fold between E9.5 and E10.5, and additionally by ∼2-fold between E10.5 and E11.5. At E9.5, cell counts in Cdan^Δ*Ery*^ and normal embryos were similar. However, by gestational day E10.5, the Cdan^Δ*Ery*^ embryos showed reduction in cell counts, compared to control embryos; the difference was statistically significant by day E11.5 (*p* < 0.002). This is consistent with the severely anemic appearance of Cdan^Δ*Ery*^ embryos (Figure 1A). These low cell counts in Cdan^Δ*Ery*^ embryos at E11.5 might indicate a role for *Cdan1* in the expansion of the circulating primitive erythroid cell population. To determine whether aberrant cell-cycle progression contributes to the reduced blood cell number observed, we cultured E9.5 peripheral blood cells *ex vivo* in medium containing bromodeoxyuridine (BrdU), and subsequently analyzed BrdU incorporation. We found that on average, 16% of wild-type erythroblasts were in G0/G1, 82% in S-phase and 1.7% in G2/M. For Cdan^Δ*Ery*^ embryos, cells in the S-phase were reduced by about 20% compared to the wild-type and increased by 50% in the G0/G1 phase. This suggests a block in S-phase entry (Figure 4B).

The apoptotic mechanism plays a relevant role in the control of erythropoiesis under physiologic and pathologic conditions (Testa, 2004). Cytospin preparations of Cdan^Δ*Ery*^ embryonic peripheral blood collected at E12.5 clearly showed apoptotic erythroblasts (Figure 4C). We therefore used AnnexinV staining to evaluate the extent of apoptosis at earlier stages. E10.5 peripheral blood cells were gated for Ter119^+^CD71^+^ erythroblasts, then analyzed for AnnexinV/7AAD staining to assess for apoptosis. Cdan^Δ*Ery*^ embryos showed an increase in both early- and lateapoptotic erythroblasts compared to littermate controls (Figure 4D). During erythroid maturation, red cell numbers are controlled by the inhibition of apoptosis, via Epo-dependent upregulation of the antiapoptotic protein Bcl-X_*L*_ (Dolznig et al., 2002). Since Bcl-X_*L*_ is a critical antiapoptotic regulator of erythropoiesis (Testa, 2004), we examined its expression levels, which was significantly reduced in primitive erythroblasts isolated from E11.5 Cdan^Δ*Ery*^ embryos (Figure 4E). Interestingly, we also found increased expression levels of the pro-apoptotic gene p53 in E11.5 Cdan^Δ*Ery*^ erythroblasts (Figure 4E). Taken together, these results indicate that the severe anemia observed in Cdan^Δ*Ery*^ embryos is caused by increased cell-death rate, as well as by cell cycle abnormalities.

### Cdan^Δ*Ery*^ Embryos Have Defects in Primitive Erythroid Differentiation

**FIGURE 5:**
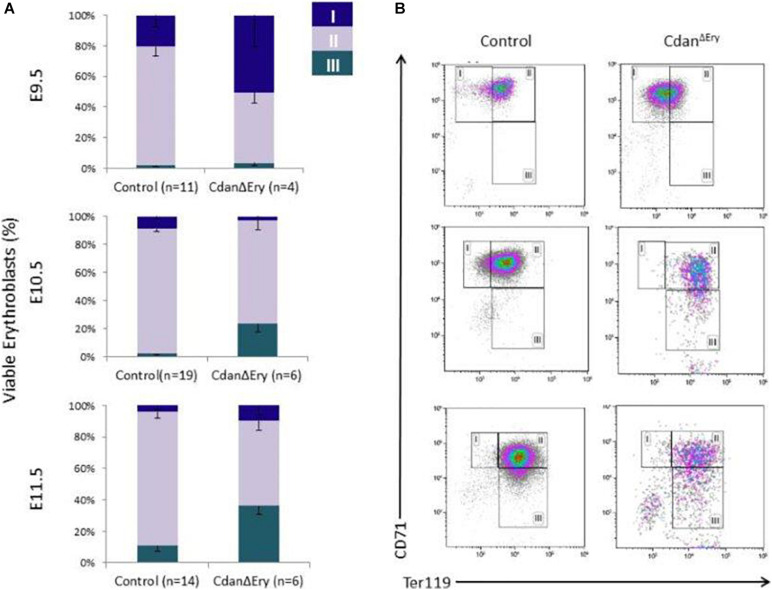
*Cdan1* is required for primitive erythroid differentiation. **(A)** Cdan^Δ*Ery*^ erythroblasts *do not* demonstrate the semi-synchronous maturation characteristic of primitive erythroblasts. Quantification of erythroid sub-populations represented as mean ± SEM. Sub-population were grouped by CD71/Ter119 surface expression according to the following: I – Ter119loCD71+; II – Ter119^+^CD71^+^; III – Ter119+CD71lo. **(B)** Representative FACS profile of Cdan^Δ*Ery*^ peripheral blood erythroblasts compared to littermate controls.

We next assessed the effect of *Cdan1* deletion on erythroid differentiation. Primitive erythroid progenitors (EryP) first emerge within the developing yolk sac at the late primitive streak stage (approximately E7.25). EryP expand in number for 48 h within the developing yolk sac, but are then rapidly extinguished by E9.0 (Palis et al., 1999). The transient appearance of EryP leads to the generation of a wave of synchronously maturing erythroid precursors (Fraser et al., 2007). The cells continue to mature in the developing circulatory system, until they expel their nuclei, around midgestation (Kingsley et al., 2004; Fraser, 2013). To monitor erythroid differentiation in these circulating primitive erythroid cells, we used both flow cytometry (FC) and morphological analysis. FC analysis of E9.5–E11.5 erythroblasts demonstrated that Cdan^Δ*Ery*^ cells are larger (based on their FSC profile, data not shown) and *do not* mature in the semi-synchronous fashion characteristic of primitive erythroblasts. While Cdan^Δ*Ery*^ E9.5 cells showed an increase in immature (Ter119^*lo*^CD71^+^) cells, the later E10.5–E11.5 erythroblasts demonstrated an increase in the more mature cell-surface phenotype (Ter119^+^CD71^*lo*^) (Figure 5). Consistent with FC results, cytospin preparations clearly show larger, more mature cells. A higher proportion of E10.5–E11.5 Cdan^Δ*Ery*^ cells have an orthochromatic cytoplasm, more condensed nuclei, and a lower nucleus-to-cytoplasm ratio (Figure 2A). We also noticed large, enucleated cells (Figure 2A, arrows), which are not expected in the circulation before E12.5 (Kingsley et al., 2004). We found 3% enucleated cells in E10.5 samples (*N* = 639 cells counted) and 7% in E11.5 (*N* = 365 cells counted), compared to less than 0.5% in control embryos (*N* = 330–367 cells counted). Together, these data suggest that in addition to their non-synchronous maturation, Cdan^Δ*Ery*^ primitive erythroblasts show accelerated terminal maturation compared to that observed in normal development.

**FIGURE 6:**
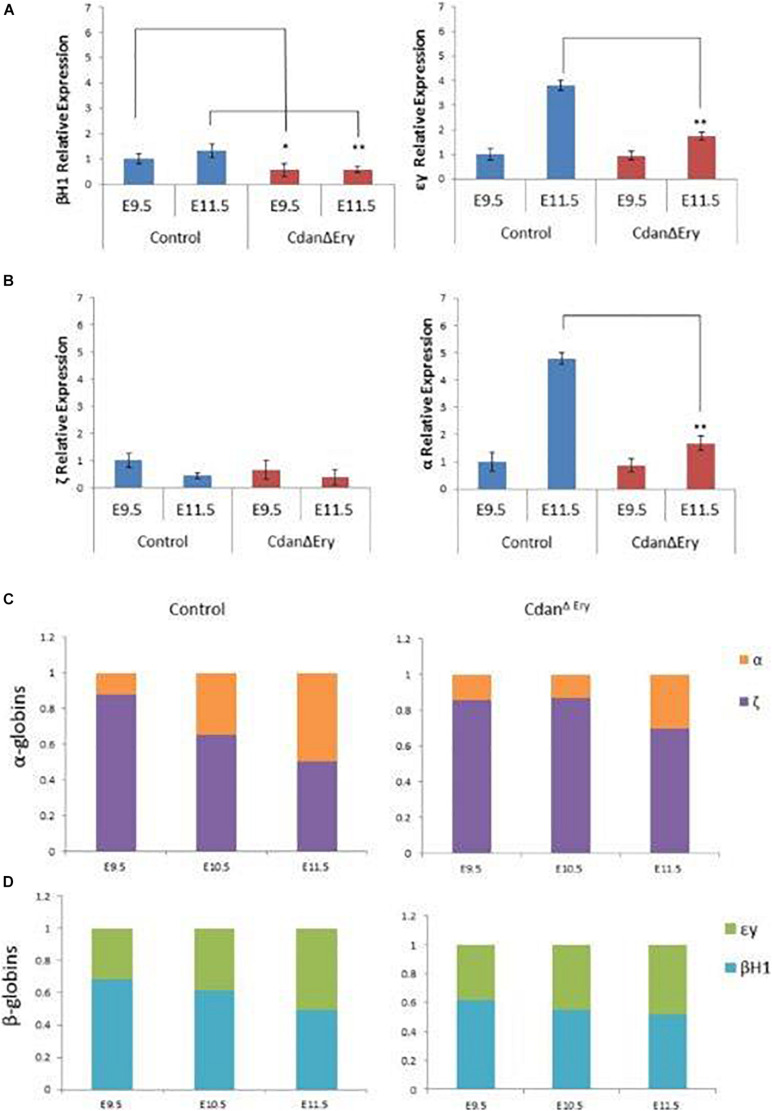
Dysregulated embryonal globin expression in Cdan^Δ*Ery*^ erythroblasts. qRT-PCR was performed with peripheral blood at E9.5 and E11.5, and normalized to Rplp0. The relative expression of embryonic β-like globins **(A)** and embryonic α-like globin **(B)** are presented as mean ± SEM (*n* = 4–6 for each genotype). The Student’s *t-*test was applied for statistical analysis. Asterisks indicate significant difference from the Control. *p < 0.01; **p < 0.001. **(C)** α-globin switch is delayed in Cdan^Δ*Ery*^ erythroblasts. Expression of ζ and α was determined by RT-qPCR. The data are displayed as the fraction of total α-like globin (mζ+mα) expression. Embryonic day is indicated. **(D)** Expression of εy, and βh1 was determined by RT-qPCR. The data are displayed as the fraction of total embryonal β-like globin (mεy+mβh1) expression.

As they mature, primitive erythroblasts accumulate large amounts of hemoglobin. The globin loci are multi-gene clusters: mice have three functional α-globins (ζ, α1, and α*2*) and four functional β-globins (βH1, εy, β1, and β2). As primitive erythroblasts mature from proerythroblasts to reticulocytes, they undergo a βH1- to εy-globin switch, up-regulate adult β1 and β2-globins, and down-regulate ζ-globin (Kingsley et al., 2006). Whereas primitive erythroblasts express all the globin genes, definitive erythrocytes express only the adult globins (α1, α2, β1, and β2). Since we observed no benzidine staining of mutant whole embryos (Figure 1B), we wanted to determine whether *Cdan1* deficiency could be dysregulating endogenous embryonic β-like and α-like globin gene expression. Thus, we performed qRT-PCR to quantify embryonic globin mRNA levels in E9.5 and E11.5 peripheral blood. Figure 6A shows reduced expression of the earlier embryonic βh1-globin in Cdan^Δ*Ery*^ E9.5 erythroblasts, and similar expression of β-like εy-globin and both α- and ζ-globin genes in control and Cdan^Δ*Ery*^ embryos at E9.5. Normally, by E11.5, the expression of εy- and α-globin genes is increased, due to extensive maturation of circulating erythroid cells. However, E11.5 Cdan^Δ*Ery*^ embryos showed significantly lower levels of embryonic β-globins, and α- globin, suggesting dysregulated globin expression (Figures 6A,B). The increase in α-globin expression over time is delayed in Cdan^Δ*Ery*^ erythroblasts, indicating a delay in α-globin switching (Figure 6C). Taken together, these results demonstrate that *Cdan1* is necessary for primitive erythroid differentiation and that its deletion dysregulates globin expression.

### *Cdan1* Deletion Interferes With Transcriptional Regulation of Primitive Erythropoiesis

**FIGURE 7:**
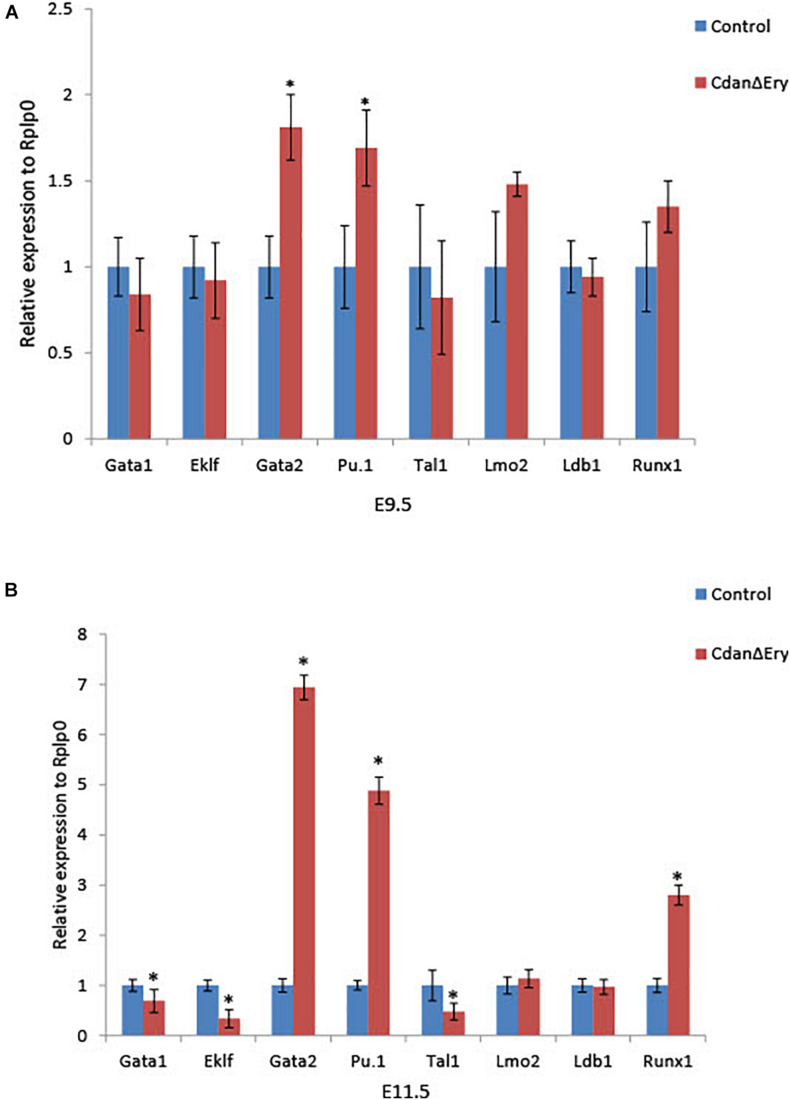
*Cdan1* ablation disrupts expression of master erythroid transcription factors. qRT-PCR was performed with RNA extracted from peripheral blood cells at E9.5 and E11.5, and normalized to Rplp0. Relative expression data are presented as mean ± SEM (*n* = 4–7 for each genotype). Student’s *t-*test was applied for statistical analysis. Asterisks indicate significant difference from the control. ^∗^*p <* 0.01 **(A)** Relative expression in E9.5 Cdan^Δ*Ery*^ circulating erythroblasts compared to littermate controls. **(B)** Relative expression in E11.5 Cdan^Δ*Ery*^ circulating erythroblasts compared to littermate controls.

Terminal maturation of erythroid cells is regulated by a host of transcriptional regulators and signaling pathways. To evaluate the effect of *Cdan1* depletion on major erythroid transcription factors, we performed qRT-PCR analyses. We examined two time points: (1) E9.5 erythroblasts which are morphologically similar to controls (as seen by light microscopy), except for the spongy heterochromatin observed by TEM; and (2) E11.5 erythroblasts, whose erythroid differentiation is already highly aberrant. E9.5 Cdan^Δ*Ery*^ erythroblasts display similar expression levels of *Eklf, Gata1, Tal1/Scl, Lmo2, Ldb1*, and *Runx1*. In contrast, expression levels of *Gata2* and *Pu.1*, which are both normally repressed in terminally differentiated erythroid cells, are elevated relative to the control (Figure 7A). Figure 7B illustrates that *Lmo2 and Ldb1* expression remained unaffected by *Cdan1* ablation in E11.5 erythroblasts. This contrasts to *Eklf*, *Gata1*, and *Tal1* whose expression levels decreased significantly in Cdan^Δ*Ery*^ E11.5 erythroblasts, and to *Gata2*, *Pu.1*, and *Runx1*, whose levels continued to increase, and to reach a level ∼3–7-fold higher than control E11.5 erythroblasts (Figure 7B). Taken together, these results show that knockout of *Cdan1* in erythroid cells results in altered expression of a number of transcription factors known to regulate terminal erythroid maturation.

### Knockdown of *cdan1* Impairs Erythropoiesis in Zebrafish Embryos

**FIGURE 8:**
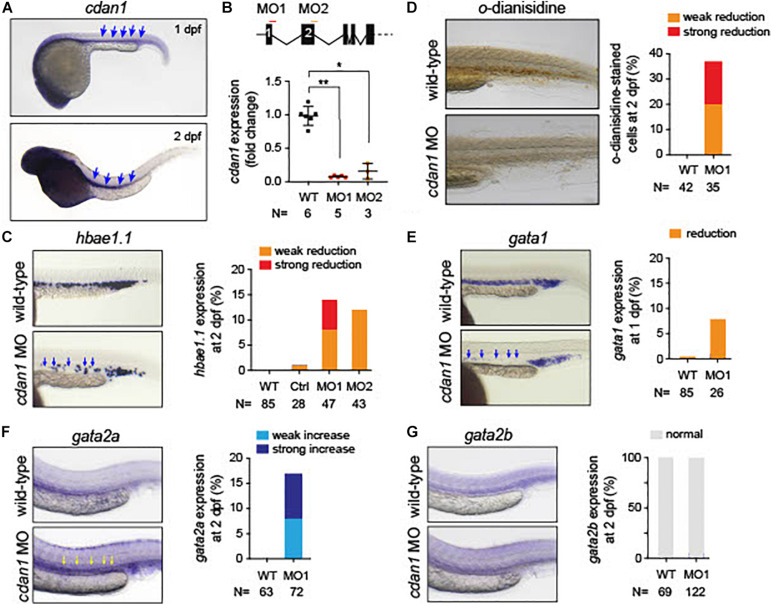
Loss of *cdan1* impairs erythropoiesis in zebrafish embryos. **(A)** Expression of zebrafish *cdan1* at 1 dpf (top) and 2 dpf (bottom). The arrows indicate the expression of cdna1 in the embryonic hematopoietic site. **(B)** Top, schematic diagram of zebrafish *cdan1* gene organization. The binding sites of two morpholinos (MO) are highlighted by red and orange lines. Note zebrafish *cdan1* gene contains 28 exons, however, only the first 5 exons are illustrated. Bottom, relative *cdan1* expression in wild type (WT) and two *cdan1* morphants illustrating that the expression level of *cdan1* was strongly reduced upon MO1 or MO2 injection. N indicates the number of biological replicates. **(C)** Expression pattern of *hemoglobin alpha embryonic 1.1*. (*hbae1.1.)* in WT and *cdan1* morphants at 1 dpf. The left panel depicts representative images of the hematopoietic tissue. The right depicts the frequency of embryos that show phenotype. Note that injection of control (Ctrl) MO showed a minor effect on *hbae1.1.* expression. **(D)** *o*-dianisidine staining in WT and *cdan1* morphants. The left panel depicts representative images of stained embryos 2 dpf. The right panel depicts the frequency of embryos showing phenotype. **(E–G)** Expression pattern of *gata1* **(E)**, *gata2a* **(F)**, and *gata2b* **(G)** in the WT and *cdan1* morphants. The left panel depicts representative images of the hematopoietic tissue. The right panel depicts the frequency of embryos that show phenotype. N indicates the number of biological samples.

Since zebrafish has been used as a model of anemia (Kulkeaw and Sugiyama, 2012), we explored the role of *CDAN1* in zebrafish embryonic erythropoiesis. First, we carried out an *in silico* analysis to identify the human ortholog gene in zebrafish. Multiple alignment and phylogenetic analysis showed high amino acid similarities over the entire coding region of the Codanin-1 protein between zebrafish, mice and humans (Supplementary Figure 3). A search in Single-Cell RNA-seq databases of zebrafish hematopoietic cells revealed that the zebrafish *cdan1* gene is predominantly expressed in erythroid cells, albeit at a low level. To examine whether *cdan1* is also expressed during zebrafish primitive erythropoiesis, we performed whole-mount *in situ* hybridization (WISH) at 1 dpf. We detected the *cdan1* transcript in the posterior intermediate cell mass, where the primitive erythropoiesis occurs (Figure 8A). Next, to examine a possible role of *cdan1* in zebrafish erythropoiesis, we used a morpholino (MO) mediated gene knockdown strategy. Specifically, two MOs targeting the *cdan1* splicing sites were designed (Figure 8B, top panel). Quantitative analysis showed that the wild-type *cdan1* transcript was decreased by about 85–93% in the MO injected embryos (hereafter called morphants) compared to uninjected wild-type counterparts. This reduction was higher in MO1 (93%) than MO2 (Figure 8B, bottom panel). To examine whether *cdan1* knockdown impairs primitive erythropoiesis, we first assessed the expression of *hemoglobin alpha embryonic 1.1* (*hbae1.1*). We found that up to 15% of morphants showed a reduction of *hbae1.1* expression in the hematopoietic tissue, compared to wild-type embryos at 1 dpf (Figure 8C, left panel). Importantly no significant difference was observed when a control MO was injected (Figure 8C, right panel). This indicates that knockdown of *cdan1* impairs primitive erythropoiesis. To further confirm this notion, we investigated the total amount of hemoglobin using *o*-dianisidine staining. Interestingly, the stain for hemoglobin was significantly reduced in the *cdan1* MO1-injected embryos at 2 dpf (37%, *N* = 35, Figure 8D). Next, we examined the expression patterns of *gata1* and *gata2* genes. Consistent with our results from mouse Cdan^Δ*Ery*^ erythroblasts, the expression of *gata1* was slightly reduced in the morphants at 2 dpf (8%, *N* = 26, Figure 8E). The zebrafish genome contains two human *GATA2* orthologs, *gata2a* and *gata2b* (Gillis et al., 2009). The former gene is expressed at a high level in hematopoietic stem cells (HSCs) and multipotent progenitors, and its expression is downregulated during erythroid/myeloid differentiation (Kobayashi et al., 2019). In contrast, the expression of *gata2b* is restricted to a subset of endothelial cells, which are required for the generation of definitive HSCs (Butko et al., 2015). WISH analysis showed noticeable induction of *gata2a* in the *cdan1* morphants, compared to the wild-type counterparts (16%, *N* = 72, Figure 8F). However, no significant difference in *gata2b* expression was observed between *cdan1* morphants and the wild-type embryos at 2 dpf (Figure 8G). Taken together, these results mirror our observations in mouse Cdan^Δ*Ery*^ erythroblasts and provide further support of the involvement of *cdan1* in primitive erythropoiesis, possibly through the regulation of transcription factors *gata1* and *gata2*.

## Discussion

CDA type I is an autosomal recessive disease associated with moderate to severe macrocytic anemia (Iolascon et al., 2013). CDA-I is caused by bi-allelic mutations in either *CDAN1* (∼80%) or *CDIN1* (∼10%) (Dgany et al., 2002; Babbs et al., 2013). The proteins encoded by both genes are ubiquitously expressed, and highly conserved. Loss of the proteins seems fatal, as no patient with bi-allelic loss-of-function variants has been identified to date. Both proteins were shown to interact directly and to bind the histone chaperone Asf1. It has also been suggested that Codanin-1 acts as a negative regulator of ASF1 function in chromatin assembly (Ask et al., 2012; Shroff et al., 2020; Swickley et al., 2020). The particular vulnerability of the erythroid lineage to defects in both proteins has not been elucidated.

To shed more light on the specific role of Codanin-1 in erythropoiesis, and to circumvent the early fatality caused by a complete knockout of *Cdan1* (Renella et al., 2011), we crossed Cdan1^*flox/flox*^ mice with a transgenic mouse strain that expresses the Cre-recombinase under regulation of the endogenous erythropoietin receptor promoter (Heinrich et al., 2004). The resulting Cdan^Δ*Ery*^ mice die in-utero at mid-gestation from severe anemia (Figure 1A).

In support of the relevance of our Cdan^Δ*Ery*^ model, erythroblasts exhibit the pathognomonic morphological features described in individuals with CDA type I (Heimpel et al., 2010). Embryonic peripheral blood collected at E10.5–E11.5 demonstrated gross dyserythropoiesis with aberrant morphological features, including bi-nucleated red blood cells and basophilic stippling. These features are similar to those observed in individuals with CDA-I (Figure 2B). Spongy or “Swiss-cheese” like heterochromatin is considered the pathognomonic feature of CDA-I. TEM analysis revealed that Cdan^Δ*Ery*^ erythroblasts exhibit spongy heterochromatin (Figure 3A), and disruption of the nuclear membrane with invagination of cytoplasmic organelles, as previously described in individuals with CDA-I (Tamary et al., 1996; Heimpel et al., 2010; Figure 3B). Of note, E9.5 cells clearly showed the disease-specific TEM phenotype, even though their gross microscopic appearance was similar to cells from control embryos (Figure 2A). This suggests that a defect in chromatin organization precedes other aberrations observed in the maturing erythroblasts.

During mouse embryogenesis, two waves of hematopoietic progenitors originate in the yolk sac. The first wave consists of primitive erythroid progenitors that arise at embryonic day E7.25, whereas the second EMP wave consists of definitive erythroid progenitors that arise at E8.25 (Palis et al., 2001), remain in the yolk sac until E11.5 when they reach the circulation and the liver and give rise to definitive erythroid, megakaryocyte, myeloid and multipotent progenitors (but not primitive erythroid progenitors). Primitive erythropoiesis starts with the transient emergence of unique erythroid progenitors (EryP) in the yolk sac at E7.25. EryP peak in number at E8.25 and those progenitors are no longer detectable at E9.0 (Palis, 2014). A semi-synchronous wave of maturing nucleated primitive erythroid precursors is then evident in the mouse embryo between E9.5 and E12.5. By E12.5, the primitive erythroblasts matured to an orthochromatic stage and cell division ceases. Primitive mouse erythroblasts produce embryonal and some adult globin genes (ζ,α, βH1, εγ, β1, and β2) and switch their hemoglobin expression as they mature from βH1 to εγ, and from ζ to α globin (Kingsley et al., 2006). Primitive erythroblasts enucleate in the mouse fetus between E12.5 and E16.5 (Kingsley et al., 2004). Transcriptional factors play a critical role in driving lineage specific cellular maturation. Disruption of each of the transcription factors *Eklf* (KLF1), *Gata1*, *Tal1/Scl*, *Lmo2*, *Ldb1*, and *Runx1* leads to a marked defect in embryonal erythropoiesis (Yokomizo et al., 2008; Palis, 2014).

We found that *Cdan1* erythroid conditional knockout mice displayed severe aberrations of primitive erythropoiesis, leading to severe anemia and death in E12.5–E13.5. Although the number of circulating primitive erythroid cells was similar to the control on day E9.5, this number subsequently decreased through terminal erythropoiesis; in E11.5 their number was 11-fold lower than that of the control (Figure 4A). This decline in terminal erythroid cells appears to be due to both increased apoptosis and a cell cycle delay. We found that both early- and late-apoptotic erythroblasts were more numerous than in littermate controls (Figure 4D). These differences were associated with increased P53 expression and decreased expression of the anti-apoptotic Bcl-X_*L*_ (Figure 4E). Additionally, cell cycle analysis revealed approximately 20% reduction of cells in the S-phase compared to the wild-type, and an increase of about 50% of cells in the G0/G1 phase. This suggests a block in S-phase entry (Figure 4B). However, this is in contrast to previous studies in CDA-I patients where erythroid S phase arrest was described (Wickramasinghe and Pippard, 1986; Tamary et al., 1996). One possible explanation for the discrepancy may be the different S phase behavior with different types of mutations. While in patients with hypomorphic mutations (as null type mutations are probably lethal) cells are able to enter S phase, with a complete knockout of codanin-1, S phase entrance may also be affected.

Due to the observed decrease in the number of cells in terminal erythroid differentiation we evaluated this stage using erythroblasts morphology and flow cytometry analysis. Cdan^Δ*Ery*^ erythroblasts displayed an asynchronous maturation pattern starting at day E10.5. This was characterized by prominent dyserythropoiesis with red blood cells, at different stages of maturation, and with a lower nucleus-to-cytoplasm ratio (Figure 2). Enucleated cells were already present at day E10.5, in contrast to the normal appearance of enucleated cells only after E12.5 (Kingsley et al., 2004). At day E12.5, red blood cells were completely destroyed, probably due to apoptosis (Figure 4C). Flow cytometry studies also demonstrated asynchronous maturation, with an increase in immature cells at E9.5, and more mature cells than the control at E10.5-E11.5. Evaluation of embryonal globin switch in Cdan^Δ*Ery*^ mice, suggested a delay of ζ to α globin switch (Figure 6C).

A number of transcription factors are known to be essential for primitive erythropoiesis. Genetic knockout models demonstrate that the transcription factors *Tal1/Scl* and *Gata1*, together with co-factors *Lmo2* and *Ldb1*, are responsible for the initiation of primitive erythropoiesis (Dore and Crispino, 2011). These factors form a large multimeric complex in erythroid cells which activates downstream targets for erythroid cell growth and differentiation. To better understand the abnormalities observed in Cdan^Δ*Ery*^ erythropoiesis, we evaluated expression levels of major transcription factors involved in erythroid differentiation. At day E9.5 significantly elevated expression levels of *Gata2* and *Pu.1* transcription factors were observed (Figure 7A). Excessively higher levels were observed at day E11.5, at which point *Gata2* and *Pu.1*, as well as *Runx1* expression, was significantly high (Figure 7B). In parallel, *Eklf*, *Gata1*, and *Tal1* expression decreased significantly (Figure 7B). Interestingly, the zebrafish cdan1 morphants mimic these findings. Whether the changes seen in transcription factors expression are a direct consequence of *Cdan1* ablation, or stem from the profound defect in chromatin organization, awaits further investigation.

In summary, we have shown, for the first time, in two animal models, that *Cdan1* is essential for primitive erythropoiesis. Further studies are indicated to investigate the mechanisms by which the absence of Codanin-1 impairs erythroid differentiation, globin synthesis and transcriptional factors expression. The two animal models we established can be used to further study the role of Codanin-1 in erythropoiesis and in the pathogenesis of CDA type I. In particular, the zebrafish model is suitable to perform high-throughput drug screening (Zon and Peterson, 2005; Uechi and Kenmochi, 2019) and will help to identify new therapeutic strategies for individuals with CDA-I.

## Data Availability Statement

The raw data supporting the conclusions of this article will be made available by the authors, without undue reservation.

## Ethics Statement

The animal study was reviewed and approved by the Animal Ethics Committee of the Faculty of Medicine in Tel Aviv University (Protocol Number 01-16-019). Zebrafish were maintained according to standard protocols and handled in accordance with European Union animal protection Directive 2010/63/EU and approved by the local government (husbandry permit 35/9185.46/Uni TÜ).

## Author Contributions

SN-L planned and performed the experiments and wrote the manuscript. OD helped in planning the experiments and performing them. NM helped with performing the experiments. AA performed the TEM work. GK started the mice experiments and followed the work. LB performed experiments with the *knock-in* mice (Cdan1^*R*1054W/R1054W^). CG performed all the zebrafish work. OS-S planned the experiments and contributed to their interpretation. HT and BM conceived and designed the study and interpreted the results and contributed to writing the manuscript. All authors approved the submitted version of the manuscript.

## Conflict of Interest

The authors declare that the research was conducted in the absence of any commercial or financial relationships that could be construed as a potential conflict of interest.
