# Supplementary material for: Cdan1 Is Essential for Primitive Erythropoiesis
Source: Front Physiol. 2021 Jun 21;12:685242. doi: 10.3389/fphys.2021.685242 (PMC8255688; doi:10.3389/fphys.2021.685242)
Supplement: Supplementary file 1 [file Data_Sheet_1.docx]

**Legend to supplemental figures:**

**Figure S1: Establishment of the *Cdan1* knockout allele**: βgal and Neo cassettes are flanked by two FRT sites (marked in green) which are excised by flippase to create the floxed allele. Exons 7-10 are flanked by two LoxP sites (marked in red), and excised by Cre recombinase to create a null allele. **SA-LacZ-pA**: splice acceptor-beta-gal-polyA; **neo**: selectable marker-neomycin; Exons marked in grey boxes. (Figure adopted from (Skarnes et al., 2011).

**Figure S2: Analyses of EpoRCre expression and recombination efficiency**.

**(A**) Spleen cells of a heterozygous Cdan^f/+;^EpoRcre mouse were sorted using magnetic CD45 beads to separate erythroid/non-erythroid populations. DNA was extracted from sorted cells and genotyped. As expected, erythroid CD45 negative cells were positive for Cre recombination. **(B**) E10.5 PB erythroblasts from a Cdan^ΔEry^ embryo showing GFP expression. **(C**) Expression of *Cdan1* in peripheral blood E9.5 erythroblasts from wild type and Cdan^ΔEry^ embryos.

**Figure S3:** **Multiple alignment of the Codanin-1 orthologues amino acid sequences.**

The alignment was generated using Clustal W. Shading indicates conservation, with, strongly conserved positions are shaded in darker colors. Codanin-1 protein from human (Hs), mouse (Mm) and zebrafish (Dr) were used for the alignment.

**Supplemental Tables:**

**Table S1:** Primers for qRT-PCR

| **Gene** | **Forward primer** | **Reverse primer** |
| --- | --- | --- |
| *mCdan1* | TCGTGCCAACTTCTCCAGTG | ACCCATCATGGCTCTGATCCT |
| *mRplp0* | AGATTCGGGATATGCTGTTGGC | TCGGGTCCTAGACCAGTGTTC |
| *mHba-a1/2* | GCTGAAGCCCTGGAAAGGAT | CAGAGCCGTGGCTTACATCA |
| *mHba-x* | TGGATTCTGTGTGGGACTAAGGC | GGGCTTGGTGGGACTGTAAGG |
| *mHbb-bh1* | GGACAGGTCTTCAGCCTCTTGA | CAGATGCTTGTGATAGCTGCCT |
| *mHbb-y* | CCAGACTTGCCATCATGGTGA | TCACCACCAACCTCTTCAACAT |
| *mLdb1* | TTGTTCCTCAAAGTCATTCAAGC | ACATCCCGATCCAGCATGGTG |
| *mGata1* | AGCCTATTCTTCCCCCAAGTTTC | CTCCACAGTTCACACATCTCTG |
| *mGata2* | CACAAGATGAATGGACAGAACC | ACAGGTGCCCGCTCTTCT |
| *mRunx1* | GACCATCACCGTCTTTACAAATC | TTGGTCTGATCATCTAGTTTCTG |
| *mEklf* | TTTTCACGCTCTGACCACTTAGC | CTTATTTCATCCCCAGTCCTTGT |
| *mPu.1* | GCTTCCCTTATCAAACCTTGTCC | CAGGCGAATCTTTTTCTTGCTGC |
| *mLmo2* | GTCTGAGGAACCCGTGGATG | ACTGGTCGATGGCTTTCAGG |
| *mTal1/Scl* | CTATAGCCTTAGCCAGCCGC | TCTCATATGGGGAGGGCCTC |
| *mP53* | GTATTTCACCCTCAAGATCC | TGGGCATCCTTTAACTCTA |
| *mBcl-xl* | GCCTTTTTCTCCTTTGGCGG | TCCACAAAAGTGTCCCAGCC |
| *z cdan1* (1) | ATCGGTGCTGCAGAGTAAAGTG | CTTTCGTCGAAGCATTAAGC |
| *z cdan1* (2) | GAGAAACGATTCGGTGTCACC | CATGGGTTAATGTCTGACTGC |
| *z bactin* | GATCTTCACTCCCCTTGTTCA | GGCAGCGATTTCCTCATC |

***m – Mouse genes; z – Zebrafish genes**

**Table S2:** Zebrafish gene-specific *in situ* hybridization probes used in this study

| **Gene** | **Accession Number/Ensembl ID** | **Nucleotides X to X** |
| --- | --- | --- |
| *cdan1* | XM_688592 | 221-806 |
| *hbea1.1* | ENSDARG00000088330 | 45-323 |
| *gata1a* | NM_131234 | 368-1222 |
| *gata2a* | ENSDARG00000059327 | 193-813 |
| *gata2b* | ENSDARG00000009094 | 89-591 |
