# Supplementary figures and images for: Cdan1 Is Essential for Primitive Erythropoiesis
Source: Front Physiol. 2021 Jun 21;12:685242. doi: 10.3389/fphys.2021.685242 (PMC8255688; doi:10.3389/fphys.2021.685242)

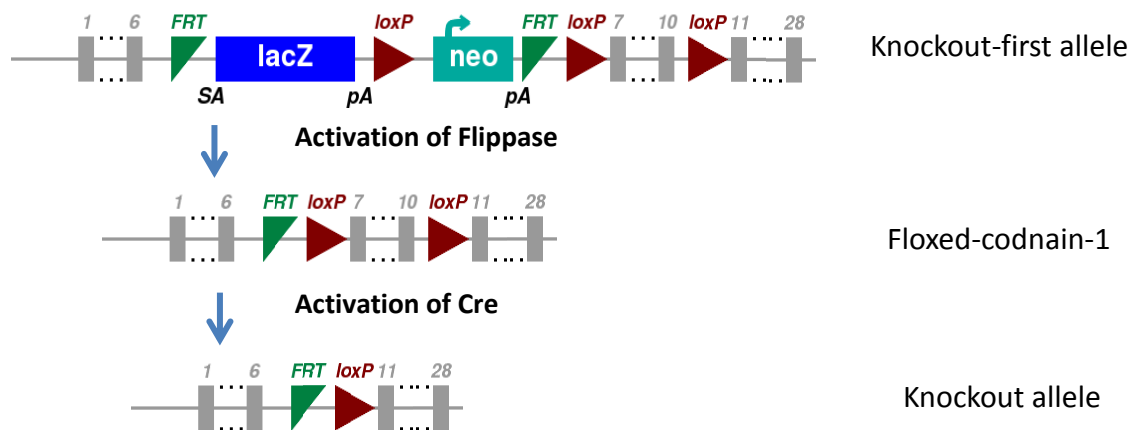

Supplement: Supplementary file 2 [file Image_1.pdf]

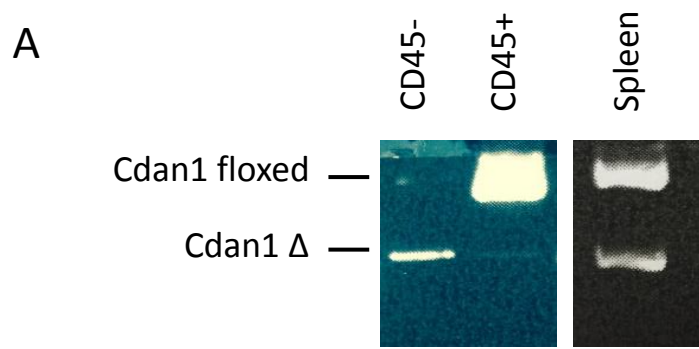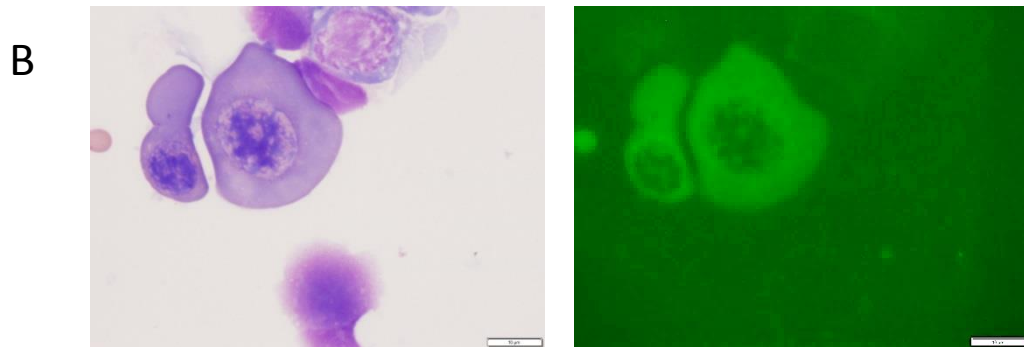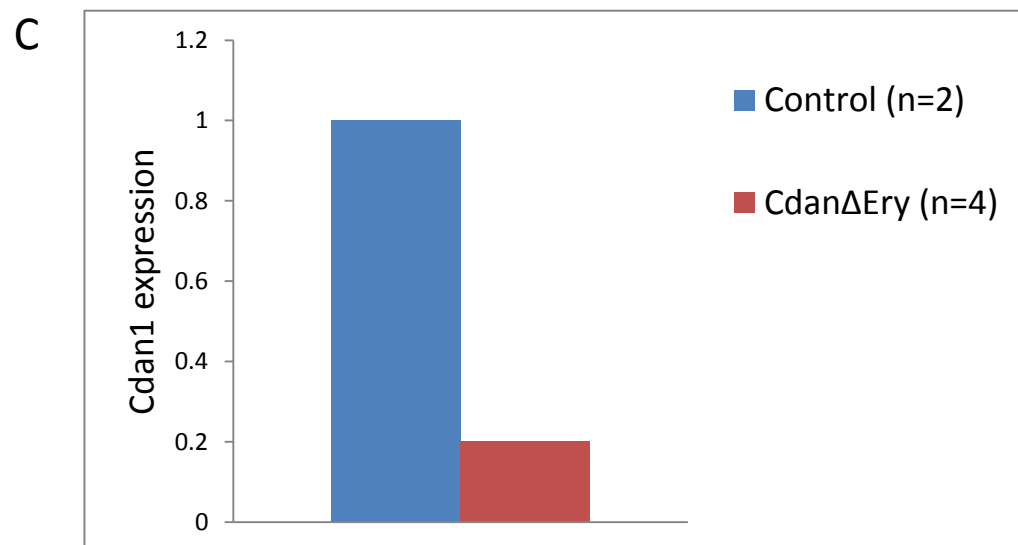

Supplement: Supplementary file 3 [file Image_2.pdf]
