# Supplementary material for: Cdan1 Is Essential for Primitive Erythropoiesis
Source: Front Physiol. 2021 Jun 21;12:685242. doi: 10.3389/fphys.2021.685242 (PMC8255688; doi:10.3389/fphys.2021.685242)
Supplement: Supplementary file 4 [file Image_3.pdf]

```
Hs CDAN1   MAAVLESLLREEVSVAAVVRWIARSTQGSEDNAGEAAALSSLRALRKEFVPFLLNFLREQSSRVLPQGPPTPAKT
Mm CDAN1   MAAVLESLLREEVPVAAAVRWIARSTPSSEDSS-EVAALSALRPLRKEFVPFLLNFLREQSSRVLPQGPSTPAKT
Dr Cdan1   MAALLESVLQSKVELSEAVLWLKGLHVCESQNF-------PSRVTKEEFVPFLLNFLRDQSSQTLTHGPATPAKT

Hs CDAN1   PGASAALPGRPGG--------PPRGSR-GARSQLFPPTEAQS---------------------------------
Mm CDAN1   PVASAALPARQGA--------PARGGR-GARSQLFPAAEPLS---------------------------------
Dr Cdan1   PSTSRSVRIQNSSERRVSKSTPVQGLRSASRVQLFSPAPFTSPGGGKNDQDTPFTGSLCLSGISALSSPSFSSVW

Hs CDAN1   --TAAEAPLARRGGRR------------RGPGP--ARERG-------GRG---LEEGVSGESLPGAGGRRLRGSG
Mm CDAN1   --AAAEAPLARRAGRR------------RGPGPGPSRERG-------GRGSGAAEEGASGESPPWAGGRKPKGSG
Dr Cdan1   SPVPRNSPSERRSAQRPSLGDFMTSPPETQPSPSAPPHRGRRRSAGFGGGTSSRQTGARGAVSEEGGGRRSRGGG

Hs CDAN1   SPSRPSLTLSDPPNLSNLEEFPPVGSVPPGPTG-TKPSRRINPTPVSEERSLSKPKTCFTSPPISCVPSSQPSAL
Mm CDAN1   SPGSPRLSLSDPPNLSNLEEFPPVGTVPPGSAGRTKPSRRINPTPVSEERSLSKPKTCFTSPPISCVPSSQPSTL
Dr Cdan1   RNDSVSPQTQVQLNFNNLEDFPPMAQPSP---IATKPSRRINPTPVSAERPNSKPKSCFTSTPLS-LPASPPKIP

Hs CDAN1   DTSPWGLGLPPGCRSLQEEREMLRKERSKQLQQSPTPTCPTPELGSP---LPSRTGSLTDEP----ADPARVSSR
Mm CDAN1   DTSPWGLGLPPGCRSLQEEREMLRKARTKQLQQSPTPASPIPESGSP---VPSRTGNLTAEP----ADPARVSSR
Dr Cdan1   EST----GSP---HNLQEERELLKRERSKLAQQTSSPLKTNSEICTPTKTVHMQGSKVTPDSQLPCPDPDKVTQK

Hs CDAN1   QRLELVALVYSSCIAENLVPNLFLELFFVFQLLTARRMVTAKDSDP--ELSPAVLDSLESPLFQSIHDCVFFAVQ
Mm CDAN1   QRLELVALIYSSCIAENLVPNLFLELFFVLQLLTARRMVATKDSDL--ESSQGALDSLDTPLFRSIHDCVFFAVQ
Dr Cdan1   SELDLLAGLYCACISENLVPCIFLEMFFVLQLLTSRGVSGSESAEQGISVCEDSPDVLEKAYLGNVHNCVYFSVR

Hs CDAN1   VLECHFQVLSNLDKGTLKLLAENERLLCFSPALQGRLRAAYEGSVAKVSLVMPPSTQAVSFQPETDNRANFSSDR
Mm CDAN1   VLEHQFQVLSYLDKGTLKLLAENERLLCFSPALQGRLRAAYEGSVAKVSLVIPPSAQAVSFQPETDNRANFSSDR
Dr Cdan1   VLEKQFELVSHLDKCTLRLLSENERVGTFSSSLKDCLAEAQDNCTAKVPPSPPLFVHSVPFQPATDNRSNFSSDK

Hs CDAN1   AFHTFKKQRDVFYEVLREWEDHHEEPGWDFEKGLGSRIRAMMGQLSAACSHSHFVRLFQKQLLQMCQSPGGAGGT
Mm CDAN1   AFHTFKKQRDVFYEVLREWEDHHEEPSWDFEKGLGSRIRAMMGQLSAACSHSHFVRLFQKQLLQMCQSPGGAGGS
Dr Cdan1   AFHTFKKQRDVFYGLLREWEDSHKEPGWDFEAALGNRVRMMVSQLTAAGSQSHFARLFQKQLVQMCKGPSVLASS

Hs CDAN1   VLGEAPDVLSMLGADKLGRLWRLQERLMAPQSSGGPCPPPTFPGCQGFFRDFILSASSFQFNQHLMDSLSLKIQE
Mm CDAN1   VLGEAPDVLNMLGADKLGRLRQLQERLIAPQSSGGPCPPPTFPGCQGFFRDFIMSASSFHFNQHLMDSLSLKIRE
Dr Cdan1   SDAPDADLLGMLGADSLGRLKRLQQRLVQPQSLIGPCPPPSFPGHQEFFKGFLQTASSCQLNQHLKDGLCQQLLK

Hs CDAN1   LNGLALPQHEPNDEDGE----SDVDWQGERKQFAVVLLSLRLLAKFLGFVAFLPYRGPEPPPTGELQDSILALRS
Mm CDAN1   LNGLRLPQHEPGDEDGE----SDMDWQGERRQFAVVLLSLRLLAKFLGFVAFLPYRGHEPPPTRELQDSILALRS
Dr Cdan1   LDEVSILGPDASPAQGEKTADGDMEQQDEKQRFSSVLLTARLLAKFLGFITFLPYQTSELP-SRDIQDAAVALRS

Hs CDAN1   QVPPVLDVRTLLQRGLQARRAVLTVPWLVEFLSFADHVVPLLEYYRDIFTLLLRLHRSLVLSQESEGKMCFLNKL
Mm CDAN1   QVPPVLDIRALLQQGLWARRAVLTVPWLVEFLSFADHIVPLLDYYRSVFTLLLRLHRSLVLSKENEGEMCFLNKL
Dr Cdan1   KSFPVLDVCTVLRNSVHRRRTILTVPWMVEFLSMLDYIGPLLPCYRTALCLLLQIYKRMVLGRG--GELCYMNQL

Hs CDAN1   LLLAVLGWLFQIPTVPEDLFFLEEGPSYAFEVDTVAPEHGLDNAPVVDQQLLYTCCPYIGELRKLLASWVSGSSG
Mm CDAN1   LLLAVLGWLFQIPTVPEDLFFLEDGQVDAFEVTTTASEHGLDSVPVVDQQLLYTCCPYIGELRKLLASWVSGSSG
Dr Cdan1   LMVAVLGWLFQIPVFPEDLFFSADLKEEMKELENKTSSQGLDTLPLVDQQLLYTCCPYLGEFRKLLAAFVAGSTS

Hs CDAN1   RSGGFMRKITPTTTTSLGAQPSQTSQGLQAQLAQAFFHNQPPSLRRTVEFVAERIGSNCVKHIKATLVADLVRQA
Mm CDAN1   RSGGFVRKITPTTTSSLGALPLQTSQGLQAQLAEAFFHNQPPSLRRTVEFVAERIGSNCVKHIKATLVADLVHQA
Dr Cdan1   KSGGLIRKITPTSAEPRGPSTTRSQQKLQVDLEQAFFHNQPPSLRRTVEFVAERVGSNCVKHIKATLVLELVRGG

Hs CDAN1   ESLLQEQLVTQGEEGGDPAQLLEILCSQLCPHGAQALALGREFCQRKSPGAVRALLPEETPAAVLSSAENIAVGL
Mm CDAN1   ESLLQEQLVARGQEGGDPAQLLESLCSQLCPHGAQALTQGREFCQRKSPTAVRALLPEETPAAVLSSAENIAVGL
Dr Cdan1   EKTLRDSL---GMEGVNTAKLNDCICAQLCDAGMQALERATRFCSENAPGAVRVLLPLETSPSVLTTAKSITTRL

Hs CDAN1   ATEKACAWLSANITALIRREVKAAVSRTLR------------AQGPEPAARGERRG--------CSRACEHHAPL
Mm CDAN1   ATEKACSWLSANITALIRREVKAAVSRMLR------------AQGPEPTARVERRG--------CSRACEHHAPL
Dr Cdan1   ATEKACSWLSSNITTLLKREWKSAFERVMKSVPSPLSVDAEEAEGLSRSQQVQKSASGLSAGSTCVPGCAHKVPL

Hs CDAN1   PSHLISEIK-------DVLSLAVGPRDPDEGVSPEHLEQLLGQLGQTLRCRQFLCPPAEQHLAKCSVELASLLVA
Mm CDAN1   PSHLISEIKFHHCSLQDVLSLAAGPRDPEEGVSPEHLEQLLNQMGQSLRCRQFLCPTAEQHLAKCSVELASLLVA
Dr Cdan1   GSEVMIEIK-------DVLSVAVGPRSDDEVFTVRQIESVLDRVGQTLGCRKRLSPVVEQMLLRCTVQLACKLVS

Hs CDAN1   DQIPILGPPAQYRLERGQARRLLHMLLSLWKEDFQGPVPLQLLLSPRNVGLLADTRPREWDLLLFLLRELVEKGL
Mm CDAN1   DQIPILGPPTQHRLERGHARRLLHMLLSLWKDDFQGPVPLQLLLSPRNVGLLADTRPREWDLLLFLLRELVEKDL
Dr Cdan1   GELPLVSS-----AEGNKRPDLLERFLVLWS--WTPAPPLHLLLTEPTLTAIFTATDRERTDYLFLIRQLIERGL

Hs CDAN1   MGRMEIEACLGSLHQAQWPGDFAEELATLSNLFLAEPHLPEPQLRACELVQPNRGTVLAQS
Mm CDAN1   MGHLEIEACLGRLNEAQWPGDFSEELSTLFRLFLAEPHLLEPQLRACELMQPNRGTVLAQS
Dr Cdan1   LGEQEVGTNWAKLSALSWPEESVEKFQQLS----LATQFSMPQL-------PNHRDLLQVSQ
```
